# FELASA accreditation of education and training courses in laboratory animal science according to the Directive 2010/63/EU

**DOI:** 10.1177/0023677218788105

**Published:** 2018-07-24

**Authors:** Marcel Gyger, Manuel Berdoy, Ismene Dontas, Martine Kolf-Clauw, Ana Isabel Santos, Mats Sjöquist

**Affiliations:** 1 Centre of PhenoGenomics, School of Life Sciences, Swiss Federal Polytechnic School of Lausanne, Switzerland; 2 BMS, University of Oxford, UK; 3 Laboratory for Research of the Musculoskeletal System, School of Medicine, National & Kapodistrian University of Athens, KAT Hospital, Greece; 4 Toxicology, Toulouse National Veterinary School, CREFRE, University of Toulouse, ENVT, France; 5 Physiology, NOVA Medical School, Universidade Nova de Lisboa, Portugal; 6 Swedish Centre for Animal Welfare, Swedish University of Agricultural Sciences, Uppsala, Sweden

**Keywords:** education, training, Directive 2010/63/EU, accreditation, mobility

## Abstract

This document describes how the Federation of European Laboratory Animal Science Associations (FELASA) accreditation addresses both the Directive 2010/63/EU and the related European Commission guidance document.

**The four EU Functions and beyond:**

FELASA accredits courses that fulfil the requirements of Functions A, B, C and D as defined by EU Directive, Article 23, as well as for designated veterinarians and specialists in laboratory animal science.

**Modularity and mobility:**

Cohesive courses for Functions and for very specific topics are accredited, but flexibility and mobility are possible: a researcher can start his/her training with one FELASA accredited course and complete other modules with another. A course organizer will deliver a FELASA certificate relating to the successfully completed modules.

**Accreditation process:**

The process consists of two major steps: (1) a review of full course documentation provided by the applicant will lead, if successful, to FELASA accreditation. The course is posted on the FELASA website as ‘FELASA accredited’ and the course provider can deliver FELASA certificates upon successful completion of the course; (2) successful accreditation is followed by an on-site course audit. In the case of a negative outcome of the audit, FELASA accreditation is withdrawn, the course is deleted from the list of FELASA accredited courses and FELASA certificates cannot be issued. To ensure that quality is maintained, continuation of accreditation requires regular revalidation.

## Résumé

Ce document décrit la façon dont l'accréditation de la Fédération des Associations européennes pour la science des animaux de laboratoire (FELASA) répond à la fois aux exigences de la Directive 2010/63/UE et à celles du document d'orientation connexe de la Commission européenne.

Les quatre fonctions définies par la Directive et au-delà : La FELASA accrédite les formations qui répondent aux exigences des fonctions A, B, C et D, telles que définies par l'article 23 de la Directive de l'UE, ainsi que des formations destinées aux vétérinaires désignés et des spécialistes de la science des animaux de laboratoire.

Modularité et mobilité : Les formations cohésives concernant des fonctions et celles très spécifiques sont accréditées, mais la flexibilité et la mobilité sont possibles : un chercheur peut ainsi débuter sa formation avec une formation accréditée par la FELASA et compléter lesautres modules dans le cadre d'une autre formation accréditée. Un organisateur de formation offrira un certificat FELASA relatif aux modules terminés avec succès.

Processus d'accréditation : Le processus comprend deux grandes étapes : (1) un examen de la documentation complète du cours fourni par le demandeur, et, si satisfaisant, l'accréditation par la FELASA. Le cours est publié sur le site web de la FELASA comme formation « accréditée par la FELASA » et le fournisseur du cours peut fournir des certificats FELASA lorsque la formation est achevée avec succès ; (2) l'accréditation réussie est suivie par un audit du cours sur place dans l'année qui suit l'accréditation. Dans le cas où cette vérification ne s'avérait pas satisfaisante, l'accréditation FELASA est retirée, le cours est supprimé de la liste des cours accrédités par la FELASA et les certificats ne peuvent plus être délivrés par le fournisseur de la formation. Afin d'assurer une qualité continue, l'accréditation doit faire l'objet d'une revalidation régulière.

## Abstract (de)

Dieses Dokument erläutert, inwiefern die EU-Richtlinie 2010/63 sowie das dazugehörige Leitliniendokument der Europäischen Kommission beim Akkreditierungsprozess der Federation of European Laboratory Animal Science Associations (FELASA) Berücksichtigung finden. Die vier Funktionen (Tätigkeiten) der EU und darüber hinaus: FELASA akkreditiert Kurse, die die Anforderungen der Tätigkeiten A, B, C und D im Sinne der EU-Richtlinie, Artikel 23, erfüllen, sowie Kurse für Tierärzte und Spezialisten für Versuchstierkunde. Modularität und Mobilität: Es werden feste Kurse für Funktionen und für sehr spezifische Themen akkreditiert, wobei entsprechende Flexibilität und Mobilität gewährt werden: So können Forscher ihre Ausbildung mit einem FELASA-akkreditierten Kurs beginnen und andere Module im Rahmen eines anderen Kurses absolvieren. Ein Kursveranstalter stellt ein FELASA-Zertifikat über die erfolgreich absolvierten Module aus. Akkreditierungsprozess: Der Prozess besteht aus zwei Hauptschritten: (1) Eine Überprüfung der vollständigen Kursunterlagen durch den Antragsteller führt bei Erfolg zur FELASA-Akkreditierung. Der Kurs wird auf der FELASA-Website als “FELASA-akkreditiert ” veröffentlicht und der Kursanbieter kann nach erfolgreichem Abschluss des Kurses FELASA-Zertifikate ausstellen; (2) die erfolgreiche Akkreditierung wird von einem Kursaudit vor Ort begleitet. Fällt der Audit negativ aus, wird die FELASA-Akkreditierung zurückgezogen, der Kurs wird aus der Liste der FELASA-akkreditierten Kurse gestrichen und FELASA-Zertifikate dürfen nicht ausgestellt werden. Zur Wahrung der Qualität muss die Akkreditierung regelmäßig verlängert werden.

## Resumen

Este documento describe cómo la acreditación de la Federación Europea de las Asociaciones de Ciencias de Animales de Laboratorio (FELASA) trata la Directiva 2010/63/UE y el documento de guía relacionado de la Comisión Europea.

Las cuatro funciones de la UE y más allá: FELASA acredita cursos que cumplen con los requisitos de las funciones A, B, C y D tal y como se define en la Directiva de la UE, artículo 23, además de ir dirigidos a veterinarios expertos y especialistas en las ciencias de animales de laboratorio.

Modularidad y movilidad: Los cursos cohesivos para Funciones y para temas muy concretos están acreditados, pero hay flexibilidad y movilidad: un investigador puede empezar su formación con un curso acreditado de FELASA y completar otros módulos con otros. Un organizador de un curso facilitará un certificado FELASA para los módulos completados satisfactoriamente.

Proceso de acreditación: El proceso consiste en dos pasos principales: (1) una revisión de toda la documentación del curso facilitada por el solicitante llevará, de completarse satisfactoriamente, a una acreditación FELASA. El curso se publica en la página web de FELASA como “Acreditado por FELASA” y el proveedor del curso puede otorgar certificados FELASA tras la finalización satisfactoria del curso; (2) a la acreditación satisfactoria le sigue una auditoría del curso in situ. En el caso de un resultado negativo de.

## Background of this document

Article 23 of the Directive on the Protection of Animals Used for Scientific Purposes 2010/63/EU (http://ec.europa.eu/environment/chemicals/lab_animals/pubs_guidance_en.htm) recognizes the importance of education and training of all persons involved with the breeding, supplying and use of laboratory animals. Annex V of the Directive identifies a list of topics to be included in education and training and the National Competent Authorities endorsed a European Union (EU) working document proposing a common education and training framework.^1^

Article 23 of the Directive recognizes competence of personnel required to carry out four Functions, designated as A (carrying out procedures on animals), B (designing procedures and projects), C (taking care of animals) and D (killing animals). These four Functions, however, differ from the Categories used previously by the Council of Europe and by the Federation of European Laboratory Animal Science Associations (FELASA) since the 1990s (see Appendix 1). Thus, the FELASA accreditation scheme was adapted to address Functions A to D as defined by 2010/63/EU (Article 23, paragraph 2) and their associated learning outcomes. In addition to these four Functions, FELASA accreditation is also available to the education and training of what we define as specialists in laboratory animal science (Specialist in LAS) – a person who may be involved in tasks described in Articles 24, 25 and 26 (fulfilling specific requirements for the welfare and care of animals, for the education and training of personnel or any other requirement for the education and training of the designated veterinarian).

## The current European education and training process

The Expert Working Group established by the Commission recommended a modular approach to the development of competencies, with defined learning outcomes^1^ (see Appendix 2). It acknowledged that the objective of initial training is to instil basic knowledge and/or understanding and is only the first step of the learning process. It involves a programme of work/study leading to specific learning outcomes, which provide basic understanding and skills appropriate to the Function.

**Figure 1.**
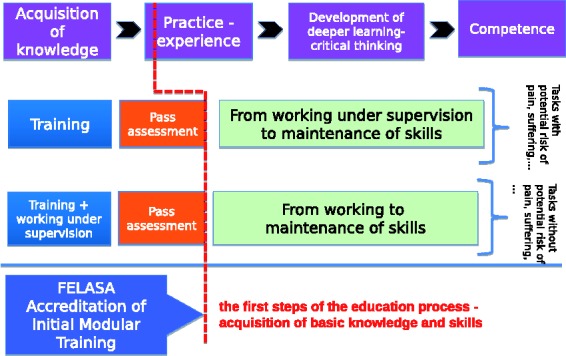
The steps of the EU education process that FELASA accredits (adapted from the European Commission guidance document, 2014).

Satisfactory completion of this initial training is followed by working with animals under supervision, leading to deeper understanding (Figure 1). This second level of training promotes the necessary *in vivo* competences for caring for and working with experimental animals in a fully responsible way and in accordance with the ‘3Rs’. This may be developed further with a programme of Continuous Professional Development.

The EU Expert Working Group recommended that persons performing one of the Functions A, B, C or D during which there is a likelihood of pain, suffering, distress or lasting harm should have completed relevant training prior to working under supervision.^1^ In other cases, the trainee could begin working under supervision before the relevant modules have been satisfactorily completed.^1^ Responsibility for correct performance of tasks always remains with the supervisor until training has been completed and the requisite competence demonstrated. Guidelines for supervision requirements are available.^2^

These requirements are reflected in different approaches to the assessment of satisfactory training and attainment of competence. The FELASA accreditation scheme addresses the first steps of the education process – acquisition of basic knowledge and skills, which are concluded by an examination (Figure 1) – and ensures their international recognition as a benchmark of high quality.

## The FELASA accreditation scheme

### Functions and modules to match specific needs of personnel

FELASA accreditation is available for courses that develop initial training leading to competency in each of the four Functions identified in the Directive. A course is defined here as a training programme consisting of one or more modules designed to provide the education and training needs of personnel dedicated to a specific Function. It may or may not have been endorsed/accredited by a National or Regional Authority or a Laboratory Animal Science Association. For a few exceptions, please see the section entitled ‘Accreditation of training for Function and for stand-alone modules’ below. Moreover, the scheme is also available for designated veterinarians and specialists in laboratory animal science (EU Directive, Articles 24, 25 and 26).

Because of its modular structure, the education scheme recommended by the EU allows the development of competence by following courses tailored to the needs of the participants. A brief description of the modules can be found in Appendix 2.

Table 1 gives a summary of the different types of modules that participants must or can attend for EU Functions A to D defined under Article 23 and illustrates how courses can be organized to provide an educational programme that covers several Functions at once. It is important to remember that the course should be relevant to the species chosen.

**Table 1.** Mapping of modules to Functions and specific tasks.

EU ID	Module description	EU Function				Species specific
		A	B	C	D^a^	
1	National legislation	C	C	C	C	
2	Ethics, animal welfare and the 3Rs (level 1)	C	C	C	C	
3.1	Basic and appropriate biology	C	C	C	C	Yes
4	Animal care, health and management	C	C	C	C	Yes
5	Recognition of pain, suffering and distress	C	C	C	C	Yes
6.1	Humane methods of killing	C	C	C	C	Yes
3.2	Basic and appropriate biology – skills	F		F	F	Yes
7	Minimally invasive procedures without anaesthesia	F	F			Yes
8	Minimally invasive procedures without anaesthesia – skills	F				Yes
9	Ethics, animal welfare and the 3Rs (level 2)		F			
10	Design of procedures and projects (level 1)	T	F			
11	Design of procedures and projects (level 2)		F			
6.2	Humane methods of killing – skills	T		T	F	Yes
20	Anaesthesia for minor procedures	T	T			
21	Advanced anaesthesia for surgical and prolonged procedures	T	T			
22	Principles of surgery	T	T			
23	Advanced animal husbandry, care and enrichment practices			T		

EU Function A: carrying out procedures on animals; EU Function B: designing procedures and projects; EU Function C: taking care of animals; EU Function D: killing animals. Level 1: At this level the trainee should describe and explain the subjects taught; level 2: at this level the trainee should show detailed understanding and should be able to critically evaluate the subjects taught.

a Module 6.3 is a stand-alone module for EU Function D (not mentioned here).

C: core modules; modules that are required for all Functions; F: function-specific (prerequisite) modules; T: task-specific modules: modules that are relevant to specific tasks within a Function.

A course provider can therefore build up a course by combining modules. The simplest course structure is the EU Function C for person taking care of animals. It comprises six core modules and one Function-specific module (Module 3.2).

The addition of the two modules on minimally invasive procedures without anaesthesia (Modules 7 and 8: theory and skills) leads to a course covering requirements for EU Function A (carrying out procedures on animals) in addition to EU Function C.

The EU Directive and particularly the European Commission guidance document^1^ allow tailoring of training required by specific tasks within a Function. This is the role of the additional task-specific modules (Table 1). Note that Module 10 (on experimental design) is a Function-specific module of EU Function B (i.e. compulsory) but can be included in an EU Function A curriculum (i.e. task specific); this choice could, for example, be specifically delivered to PhD students carrying out research under supervision of the principal investigator.

A course for EU Function B could be built as a stand-alone course that is entirely theoretical and does not include skills training. However, it is easy to conceive, as a way of example, that a course organizer may propose a programme combining both the required elements for EU Functions A (persons carrying out animal experiments) and B (persons responsible for designing experiments). Thus, a person aiming for EU Function B could decide to attend the additional elements required for EU Function A. The benefit of a combined training is that such a person would be better informed regarding the practicalities of performing procedures and the necessity of adopting best practices in the design of projects than a person with only EU Function B education. A course based on such a unified programme would deliver FELASA accredited certificates with relevant modules for EU Functions A *or* B for persons who have successfully completed the relevant parts of the programme, or a certificate for EU Functions A *and* B for those who have completed both.

If only EU Function B courses are delivered, it is possible to cover the requirements of EU Function C by adding Module 3.2 to the EU Function B course.

Humane killing of animals is recognized as a separate Function under the EU Directive (Function D). A stand-alone module (6.3) can fulfil the training requirement for those who carry out euthanasia only. In practice, however, this Function is often carried out alongside others such as caring for animals (EU Function C) or carrying out procedures (EU Function A). In these cases, it is recommended to add Module 6.2 to those of EU Function A or C to fulfil the training requirements of EU Function D as well (Table 1).

### Accreditation of training for Function and for stand-alone modules

The FELASA accreditation scheme is based on the modules set out in the document endorsed by the National Competent Authorities (European Commission guidance document;^1^ see Appendix 2) but for these to be accredited they are expected to be part of a broader programme fulfilling the requirements of a specific Function. Thus, a FELASA accredited programme will be expected to consist of at least: a) the ‘Core Modules’, b) the modules that are prerequisite to that Function (see ‘Function-specific modules (prerequisite)’ – Table 1/Appendix 2), and c) cover at least one species or a group of species. These constitute the minimum requirements that are expected to be covered before a course application can be accepted for FELASA accreditation.

However, the FELASA Accreditation Board for Education and Training (FELASA E&T Board) will accept applications for a few stand-alone modules. These include:

- *Function B* modules 9, 10 or 11.
- *Task-specific* modules 20, 21 and 22, or 23.
- *Other additional modules* 50 and 51.
- *Stand-alone* module 6.3.

See Table 1/Appendix 2 for a description of each module.

The FELASA E&T Board will not apply the stand-alone module accreditation policy to any *core* module.

### Roles other than the EU Functions A, B, C and D described in the Directive

In addition to the four Functions mentioned above, the FELASA accreditation covers courses for other roles; the Designated Veterinarian and the Specialist in LAS are already part of its portfolio. Courses for Project Evaluators will be potential candidates for accreditation.

**Table:** 

Module 1	‘National legislation’ with additional Learning Outcomes as described in Module 24 (24.1–24.5);
Module 9	‘Ethics, animal welfare and the 3Rs’ (level 2) with additional Learning Outcomes as described in Module 24 (24.6–24.12);
Module 10	‘Design of procedures and projects’ (level 1);
Module 50	‘Introduction to the local environment’.

The European Commission guidance document^1^ gives the list of learning outcomes for a Designated Veterinarian. In addition to Module 24 it includes other modules as described below:

### Mobility of students and FELASA accredited course attendance

The FELASA scheme accredits courses or training programmes, not persons. The rules governing the accreditation of individuals are, of course, the responsibility of relevant Competent Authorities, not of FELASA. To facilitate access to training as well as mobility of personnel, persons undertaking training for a specific Function will be able to combine FELASA accredited courses in different institutions to gain qualification. In this scenario, students can start training at one institution and complete their training by taking part in another FELASA accredited course at a different institution. Organizers of FELASA accredited courses will be able to deliver a FELASA certificate for the specific module(s) of the course completed by the student.

### Entry qualifications

Although there are no specific entry qualifications for three of the four Functions, the EU Directive indicates that a person with a Function B responsibility ‘…shall have received instruction in a scientific discipline relevant to the work being undertaken and shall have species-specific knowledge…’ (Article 23, 2). More specifically the EU Commission guidance document (p. 7),^1^ recommends that the person designing procedures and projects should have received a university degree or an equivalent degree in the relevant scientific discipline.

The FELASA E&T Board regards a bachelor’s degree or equivalent as the minimum entry qualification for a Function B course. As a consequence, when a course covers several Functions, including among them Function B, entry qualification criteria for that Function must be satisfied.

If there is no legally defined entry qualification, the course organizer has to ensure that, for each module taught, all students should attain the minimal level of understanding of the learning outcomes at the end of the course, irrespective of their prior level of knowledge.

### Manual skills training using live animals

The issue of the use of live animals in laboratory animal science education and training courses has been debated for many years. When launching its accreditation scheme in 2003, FELASA issued the following statement: ‘It can be concluded that the use of live animals in courses for scientists can contribute both to the development of skills and enhancing attitude, when the teaching is of a high standard and stresses the moral dimension…’.^3^ We believe that this statement remains valid today.

The EU commission has made it clear that the Directive allows the use of live animals for education and training and describes under which conditions the use of live animals is justified (EU Commission guidance document, pp.31–32^1^). Such justification is moreover substantiated by past experiences. The FELASA E&T Board has accumulated information with the accreditation of courses under the previous FELASA category scheme. Review of past annual reports and audits indicates that students’ assessments of courses place the manual skills training using live animals among the preferred parts of the course; very often, students ask for more practical sessions. Moreover, the skill acquisition process is performed in a pedagogic and educational environment where theoretical concepts taught during the lectures are directly translated in best practices during skills training; this might not be the case in an on-the-job set-up carried out in the research laboratory (see also Carlsson et al.^4^). The FELASA E&T Board’s view is that the provision of contextual understanding in educational courses gives a solid basis for building up skills for future research with live animals. This results in a positive effect on scientific integrity and prevents avoidable compromises to animal welfare.

Not all courses use live animals for skills training. However, the FELASA E&T Board considers that at least handling and restraint of live animals (EU Module 3.2) should be mandatory. In order to be able to guide students to treat animals humanely and to assess attitude, no alternative to working with live animals exists today. Prior to learning how to perform procedures on animals, certain manual skills could be taught non-invasively, such as handling needles, syringes, or suturing and others. Further, in addition to such training using alternatives, the FELASA E&T Board recommends that, whenever possible, training in minimally invasive procedures (Module 8) is carried out on live animals under specific conditions that are described below:

1. The applicant must provide a proof that the manual skills training has been submitted to an ethical evaluation process and has been accepted by the competent authorities.
2. As recommended by the European Commission guidance paper,^1^ the applicant should demonstrate that he/she applies a tiered approach from non-animal alternatives to cadavers and live animals, depending on the procedure. The severity of the procedures should be non-recovery or mild. The approach should demonstrate the effective implementation of the 3Rs.
3. The training of manual skills should address housing and basic care of animals, include handling and restraint of conscious animals and some minimally invasive procedures on anesthetized animals.
4. The animals to be used for skill training should come whenever possible from surplus stocks or from the re-use of animals according to the EU Directive regulations. Their fate at the end of the training should be indicated. The room where animals are housed should be clearly separated from the training rooms in order to minimize the stress to the animals. Euthanasia should be performed in a separate room.

The teaching environment and guidance of students should not be neglected. Rooms and space should be appropriate for skills training. A detailed guide should be provided beforehand to students. The ratio between number of tutors and number of students should not exceed one tutor for five trainees. Waste and personal protection utilities and equipment should be available and clearly marked. Protection against laboratory animal allergies should be included.

### Certificate format and content

The certificate should include sufficient details of what has been learnt in order to promote transparency, which will facilitate movement from one institution to another and/or acceptance by a different competent authority. Thus, the certificate should include the FELASA logo, course ID, participant’s details, modules completed and a course email address (in case complementary information is requested). Animals that have been handled as part of the practical parts of the course should be listed by the level of the species (e.g. rat, mouse, zebrafish, medaka) rather than as a group of species (e.g. rodents, fish) whenever possible. The teaching of the theoretical part, however, can cover either a single species or a group of species at the discretion of the course organizer as long as this is listed in the certificate. Thus, it is possible to cover a group of related species as part of the theory, and focus the practical training on the relevant species. In the case of a future change of animal model from one rodent species to another, for example, the researcher can receive specific hands-on training on the new rodent species in the institution without needing to attend additional lectures.

## The FELASA accreditation process

**Figure 2.**
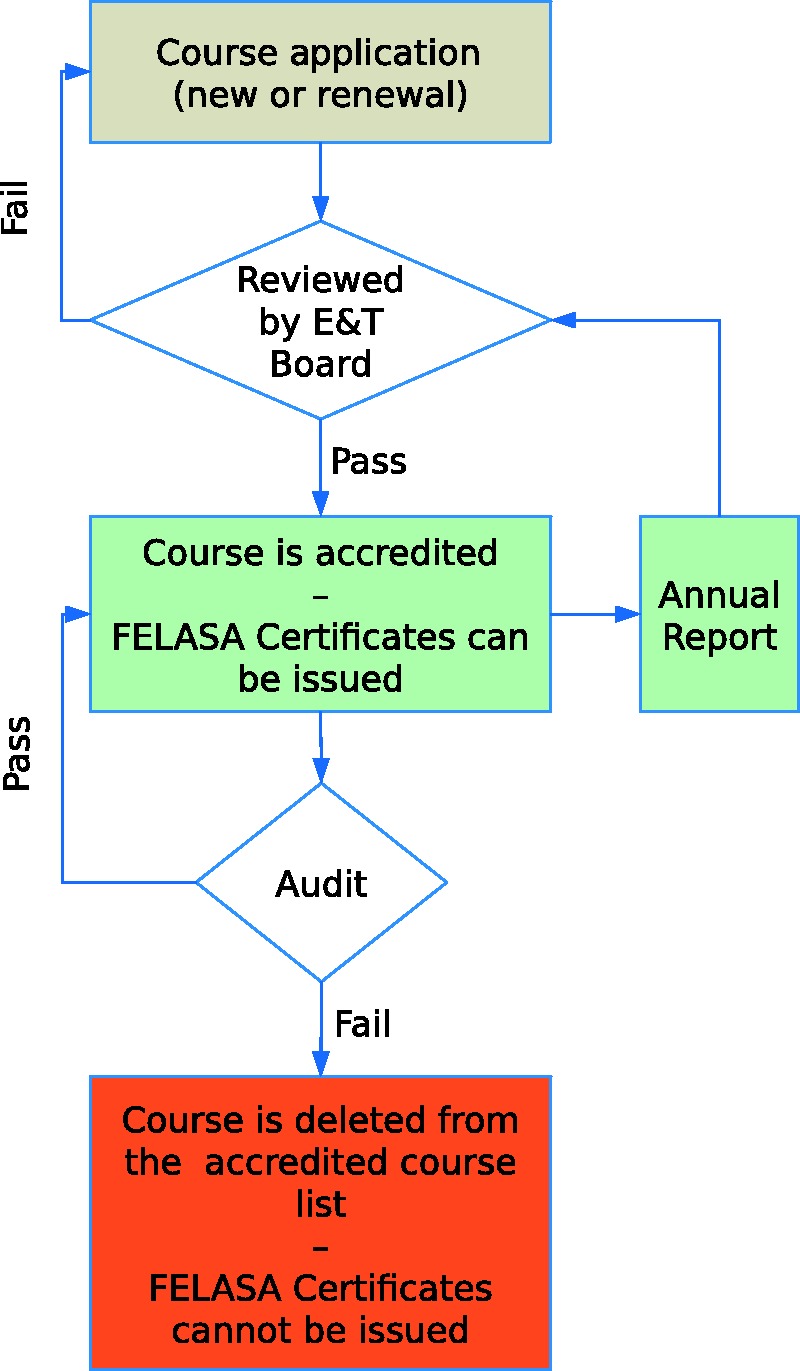
The FELASA Education and Training Accreditation Process. FELASA: Federation of European Laboratory Animal Science Associations. E&T Board: FELASA Accreditation Board for Education and Training.

In order to combine flexibility of training and speed of process, the FELASA accreditation scheme is based on a two-stage approach based on verification of course quality (Figure 2).

### Written submission

Course organizers seeking accreditation are required to complete an application form obtainable from the FELASA Secretariat.

This form seeks information about the applicant and the institution where the course is held. It asks for confirmation that the course has been run in its present form at least once; the frequency with which it is held; the number of students and admission qualifications when appropriate (see above for EU Function B); whether the use of living animals has been authorized by competent authorities and whether the course is recognized as part of a formal educational programme. Applicants must provide information about course structure and topics, practical classes and how the learning outcomes are covered, the way in which knowledge and competency are assessed and the results of those assessments. The credentials of teaching staff must also be provided along with details of how students are able to comment on the quality of the learning experience.

On the basis of this written submission, the FELASA E&T Board verifies that the training delivered meets the requirements of the relevant EU Function,^1^ reviews the mix of teaching methodology and the use made of supporting materials, such as course notes and recommended reading, and ensures that the objectives of the course conform with relevant national laws, recommendations and guidelines with official supporting documents where appropriate. Consideration is also given to the method of student assessment, the range and depth of evaluation of learning outcomes and the way in which practical skills are assessed. Course organizers must have implemented a mechanism by which students can reflect and report on their learning experience, and must provide examples of course evaluations to show that the course is balanced, effective and worthwhile. Copies of all examination papers, all student evaluation results and all teaching materials are not requested at this stage.

If the information provided is judged satisfactory, the course will be given accreditation.

Following the decision, an accreditation contract is to be signed between FELASA and the Institution that will issue the training certificates. The FELASA certificate is to be distributed to every participant who fulfilled the requirements to enter and attend the course and who passed the examination. There is only one kind of FELASA accredited certificate delivered. The course is posted on the FELASA website as ‘FELASA accredited’.

### Audit

Since the FELASA E&T Board implemented a systematic programme of course audits in 2006^5^ it has become apparent that assessment of course quality based only on written evidence may lack robustness and can on some occasions be misleading. The FELASA E&T Board therefore audits all courses whilst they are running. This enables the gathering of independent first-hand information about the quality of the learning environment.

The audit consists of a visit lasting usually one or two days by two FELASA auditors. The board requests documentary materials before the visit and a list of materials that will be examined during the audit. The latter includes examination papers and written student course evaluations, as well as notes of teachers’ meetings, records of course attendance, copies of course materials provided to the students, checklists, including teaching aids and those used for practical training, and a list of certificates issued. In addition to reviewing these documents, the auditors attend lectures and practical sessions included in the accredited programme. The auditors interview students who have already completed the course and supervisors, as well as students and teachers of the current course and others responsible for course organization and delivery.

At the end of the audit, the auditors submit a report to the FELASA E&T Board. This leads to the decision on whether the course maintains accreditation with or without changes, or if accreditation is required to be suspended until crucial changes are incorporated.

The audit is a crucial part of the accreditation. The FELASA E&T Board regards it as the most reliable method of establishing the quality of the student learning experience. It is inevitably a formal process, but it is also an opportunity for an open and constructive discussion on LAS training. Course organizers often report that they have found the audit inevitably formal but surprisingly enjoyable and an opportunity to talk about issues with like-minded people.

The FELASA E&T Board reserves the right to conduct audits, other than those involved in the accreditation process, subject to the provision of at least 10 days’ advance notice.

The FELASA E&T Board publishes a list of courses that have been FELASA accredited on the FELASA web page (www.felasa.eu).

### Appeal procedure

An applicant has the right to appeal against a decision made by the FELASA E&T Board by writing to the FELASA President within 15 days of receiving written rejection.

### Confidentiality

The FELASA accreditation process is conducted in strict confidentiality by all those involved. All communication with the applicant goes through the FELASA Secretariat.

## Requirements for maintaining FELASA accreditation

Accreditation for programmes of training delivered over a period lasting less than one year will normally be valid for five years. Courses for Functions A, B, C and D fall into this category. For courses lasting over a year, for example, courses for laboratory animal specialists, accreditation will be valid for 10 years.

Organizers of FELASA accredited courses must submit an annual report, confirming adherence to conditions specified on the application or providing explanations for changes. Further details and a report template are available from the FELASA Secretariat.

Significant changes of content from the programme approved (and not pre-advised and approved by the E&T Board), under-staffing for course delivery, inappropriate content or teaching methodology, concerns about animal welfare, occupational health issues etc. could be sufficient cause for the withdrawal of FELASA accreditation.

It is expected that course organizers will continually evaluate the content format and teaching methodology of the programme and, where appropriate, introduce modifications to improve the quality of the learning experience in light of comments from participants. In many cases, new ideas should be evaluated by introduction on a trial basis, assessed by teachers and participants and reported in the next annual report.

If it is proposed to introduce major changes to a FELASA accredited course, a detailed proposal should be submitted to the FELASA secretariat before these are implemented, so that the FELASA E&T Board can give them prior consideration and, if appropriate, approval. This requirement refers to changes of course structure and/or delivery process and is independent of the obligation to submit an annual progress report.

### Renewal

FELASA accreditation will lapse at the end of each accreditation period. If continued accreditation is required, course organizers must submit a new application in accordance with the requirements for accreditation at least six months before the existing accreditation expires. During the renewal process the course will be entitled to retain its status of accreditation.

If the first or the renewal audit reveals major problems leading potentially to the cancelling of the accreditation, the FELASA E&T Board will decide whether the application is rejected or, if satisfactory changes have been implemented, that accreditation may be continued. A second audit will then confirm accreditation of the course if the changes have addressed the shortcomings (Figure 2). In the case of a second audit being necessary, the course organizer will have to pay the costs of the auditors.

## Fee policy

The FELASA accreditation programme is a non-profit making venture, but is required to be financially self-supporting. Applications for accreditation will be processed only after an application fee has been received. The application fee is independent of the number of courses given annually and is non-refundable, irrespective of the outcome of the accreditation application. The FELASA E&T Board will propose a scale of fees annually to the FELASA Board of Management.

For courses given in Europe, the fees for the duration of the accreditation period include the cost of the audit. In the case of an audit revealing major failures in the course quality and a second audit being required, the costs of the second audit are covered by the course organizer.

For courses offered in countries outside Europe, the fee for accreditation or renewal is identical to that for European courses, but does not include the cost of the audit, which will be levied as an additional cost.

An annual fee plus a small fee for each student certificate issued during that year for the accredited course is payable for the duration of the accreditation. This annual fee is payable even if the course has not been held during that year. The current fees are listed on the FELASA website.

In the event of a course wishing to opt out of accreditation status, the remaining annual fees for the duration of the accreditation will still be payable.

## Appendix 1. The FELASA Categories and Directive Functions: An historical reminder

Three out of the four former FELASA Categories^6–10^ correspond roughly to three of the Functions defined in the Directive 2010/63/EU (Table 2) although some additional adjustments are clearly needed, for example, learning outcomes instead of syllabus, recommendations for FELASA Category A^8,10^ do not match the current Directive proposals for Function C.

**Table 2.** Comparison of the Federation of European Laboratory Animal Science Associations (FELASA) Categories and the Functions identified in Directive 2010/63/EU.

FELASA Category	Role	Directive EU Function
A	Persons taking care of animals	C (may include D)
B	Persons carrying out animal experiments	A (may include C/D)
C	Persons responsible for directing animal experiments	B (may include A/C/D)
D	Specialists in LAS	Refer to Articles 24, 25, 26
–	Persons killing animals	D

LAS: laboratory animal science.

FELASA Category D^11^ does not map directly into one of the four Directive Functions. However, it may be applicable partly to ‘persons overseeing the welfare and care of the animals, ensuring adequate information specific to the species housed available to the staff, and ensuring that the staff has the required competences’ (Article 24), to ‘designated veterinarians with expertise in laboratory animals medicine’ (Article 25) and to ‘persons responsible for the welfare and care of the animals’ (Article 26).

### Appendix 2. Modules considered as minimum training necessary before persons are allowed to carry out a Function (A to D)

These modules have been endorsed by the National Competent Authorities for the implementation of Directive 2010/63/EU on the protection of animals used for scientific purposes (see European Commission guidance document^1^ for explanation and details of learning outcomes).

#### Core modules – Functions A, B, C and D

1    National legislation;
2    Ethics, animal welfare and the 3Rs (level 1);
3.1    Basic and appropriate biology – species specific (theory);
4    Animal care, health and management – species specific (theory);
5    Recognition of pain, suffering and distress – species specific;
6.1 Humane methods of killing (theory).

#### Function-specific (prerequisite) modules – Function A: Carrying out procedures on animals

3.2   Basic and appropriate biology – species specific (practical);
7    Minimally invasive procedures without anaesthesia – species specific (theory);
8    Minimally invasive procedures without anaesthesia – species specific (skills).

#### Function-specific (prerequisite) modules – Function B: Designing procedures and projects

7  Minimally invasive procedures without anaesthesia – species specific (theory);
9  Ethics, animal welfare and the 3Rs (level 2);
10 Design of procedures and project (level 1);
11 Design of procedures and project (level 2).

#### Function-specific (prerequisite) modules – Function C: Taking care of animals

3.2  Basic and appropriate biology – species specific (practical).

#### Function-specific (prerequisite) modules – Function D: Killing animals

3.2  Basic and appropriate biology – species specific (practical);
6.2  Humane methods of killing (skills).

Alternatively:

6.3 Stand-alone module for Function D (only).

##### Additional task-specific modules

20 Anaesthesia for minor procedures;
21 Advanced anaesthesia for surgical or prolonged procedures;
22 Principles of surgery;
23 Advanced animal husbandry, care and enrichment practices;
24 Designated Veterinarian;
25 Project Evaluator.

#### Other additional modules

50 Introduction to the local environment (establishment) for persons taking specific roles under the Directive;
51 Information provision and retrieval.

## References

[1] European Commission guidance document. National Competent Authorities for the implementation of Directive 2010/63/EU on the protection of animals used for scientific purposes. A working document on the development of a common education and training framework to fulfil the requirements under the Directive. Brussels, http://ec.europa.eu/environment/chemicals/lab_animals/pdf/guidance/education_training/en.pdf (2014, accessed 4 January 2018).

[2] Jennings M and Berdoy M (eds). LASA 2016 guiding principles for supervision and assessment of competence as required under EU and UK legislation. 2nd ed. A report by the LASA Education, Training and Ethics Section, http://www.lasa.co.uk/wp-content/uploads/2016/09/LASA_supervision_and_competence_2016.pdf (2016, accessed 4 January 2018).

[3] Ritskes M and Hau J. FELASA statement on the use of live animals in teaching and training, http://www.felasa.eu/?ACT=43&file_id=PMh7rM%2BTYA5nKny4HER14UJGh2%2F%2FyBpeRdWZqt7i9sbjNTwpHEYFfLvgPRCP6mPNxKrtdwH3zyaSQiNl3zG48Q%3D%3D&access=YPCn9I2eJRBxlXNvCmqNBnXwgTDnfCvMV%2BtAgOMTqdGtDgHVEh4FIk5nhhpthPuG5PJFVH4vpLlMx6FgPxxSGQ%3D%3D (2003, accessed 4 January 2018).

[4] Carlsson H-K Hagelin J Höglund AU et al. Undergraduate and postgraduate students’ responses to mandatory courses (FELASA category C) in laboratory animal science. Lab Anim 2001; 35: 188–193. 11315170 10.1258/0023677011911462

[5] Nevalainen T Blom HJ Guaitani A et al. FELASA recommendations for the accreditation of laboratory animal science education and training. Lab Anim 2002; 36: 373–377. 12396280 10.1258/002367702320389035

[6] Council of Europe. European Convention for the Protection of the Vertebrate Animals used for Experimental and other Scientific Purposes. Article 26, Strasbourg, http://conventions.coe.int/Treaty/en/Treaties/Word/123.doc (1986, accessed 4 January 2018).

[7] Council of Europe. Resolution on Education and Training of Persons Working with Laboratory Animals adopted by the Multilateral Consultation on 3 December 1993, Strasbourg, http://www.coe.int/t/e/legal_affairs/legal_co-operation/biological_safety_and_use_of_animals/laboratory_animals/Res%20training.asp (1993, accessed 4 January 2018).

[8] FELASA recommendations on the education and training of persons working with laboratory animals: Categories A and C. Reports of the Federation of European Laboratory Animal Science Associations Working Group on Education accepted by the FELASA Board of Management. Lab Anim 1995; 29: 121–131. 7602998 10.1258/002367795780740177

[9] Nevalainen T Dontas I Forslid A et al. FELASA recommendations for the education and training of persons carrying out animal experiments (Category B). Report of the Federation of European Laboratory Animal Science Associations Working Group on Education of Persons Carrying out Animal Experiments (Category B) accepted by the FELASA Board of Management. Lab Anim 2000; 34: 229–235. 11037115 10.1258/002367700780384672

[10] Weiss J Bukelskiene V Chambrier P et al. FELASA recommendations for the education and training of laboratory animal technicians: Category A. Report of the Federation of European Laboratory Animal Science Associations Working Group on Education of Animal Technicians (Category A) accepted by the FELASA Board of Management. Lab Anim 2010; 44: 163–169. 20427379 10.1258/la.2010.010004

[11] Nevalainen T Berge E Gallix P et al. FELASA guidelines for education of specialists in laboratory animal science (Category D). Report of the Federation of European Laboratory Animal Science Associations Working Group on Education of Specialists (Category D) accepted by the FELASA Board of Management. Lab Anim 1999; 33: 1–15. 10759386 10.1258/002367799780578561

